# Circadian Disruption across Lifespan Impairs Glucose Homeostasis and Insulin Sensitivity in Adult Mice

**DOI:** 10.3390/metabo14020126

**Published:** 2024-02-16

**Authors:** Tracy K. Her, Jin Li, Hao Lin, Dong Liu, Kate M. Root, Jean F. Regal, Emilyn U. Alejandro, Ruifeng Cao

**Affiliations:** 1 Department of Integrative Biology and Physiology, University of Minnesota Medical School, Minneapolis, MN 55455, USA; herxx397@umn.edu; 2 Department of Biomedical Sciences, University of Minnesota Medical School, Duluth, MN 55812, USA; lijin0213@126.com (J.L.); hao.lin2022@rutgers.edu (H.L.); cyyzld@gmail.com (D.L.); ande4808@d.umn.edu (K.M.R.); jregal@d.umn.edu (J.F.R.); 3 Institute of Neuroscience and Translational Medicine, College of Life Science and Agronomy, Zhoukou Normal University, Zhoukou 466001, China; 4 Department of Neuroscience and Cell Biology, Robert Wood Johnson Medical School, Rutgers University, Piscataway, NJ 08854, USA; 5 Spencer Center for Vision Research, Department of Ophthalmology, Byers Eye Institute, Stanford University School of Medicine, Palo Alto, CA 94304, USA; 6 Department of Neurology, Robert Wood Johnson Medical School, Rutgers University, Piscataway, NJ 08854, USA

**Keywords:** circadian disruption, glucose, insulin, obesity, mice

## Abstract

Circadian rhythm disruption is associated with impaired glucose homeostasis and type 2 diabetes. For example, night shift work is associated with an increased risk of gestational diabetes. However, the effects of chronic circadian disruption since early life on adult metabolic health trajectory remain unknown. Here, using the “Short Day” (SD) mouse model, in which an 8 h/8 h light/dark (LD) cycle was used to disrupt mouse circadian rhythms across the lifespan, we investigated glucose homeostasis in adult mice. Adult SD mice were fully entrained into the 8 h/8 h LD cycle, and control mice were entrained into the 12 h/12 h LD cycle. Under a normal chow diet, female and male SD mice displayed a normal body weight trajectory. However, female but not male SD mice under a normal chow diet displayed glucose intolerance and insulin resistance, which are associated with impaired insulin signaling/AKT in the skeletal muscle and liver. Under high-fat diet (HFD) challenges, male but not female SD mice demonstrated increased body weight gain compared to controls. Both male and female SD mice developed glucose intolerance under HFD. Taken together, these results demonstrate that environmental disruption of circadian rhythms contributes to obesity in a sexually dimorphic manner but increases the risk of glucose intolerance and insulin resistance in both males and females.

## 1. Introduction

Circadian rhythm refers to the approximate 24 h rhythmicity that is found in a variety of biological processes. It is an evolutionarily conserved property that exists in almost all living organisms. This property enables animals to predict and prepare for upcoming environmental changes and align their physiology, metabolism, and behavior with the time of day [1]. The synchronization of internal physiology with the external environment is important for achieving optimal physiological efficiency to survive in the natural environment [2]. The hypothalamic suprachiasmatic nucleus (SCN) is the master clock located in the brain that is responsible for the regulation and modulation of the mammalian circadian rhythm [3,4,5]. In addition to the SCN, various peripheral tissues such as the liver, skeletal muscle, adipose tissue, and the endocrine pancreas possess their own intrinsic clocks, coordinating a range of specialized functions within each tissue [6,7,8].

In mammals, including humans, the circadian rhythm is endogenously driven by a dozen so-called “clock” genes. BMAL1 forms heterodimers with CLOCK or NPAS2 and binds to the e-box enhancers located at the promoter regions of Period1/2/3 and Cry1/2 and drives their gene expression. When Per and Cry are translated into proteins, they form protein complexes and accumulate in the cytosol to a certain level. Then the PER/CRY complexes will translocate into the cell nucleus, interact with CLOCK/BMAL1, and inhibit their own gene transcription, thus forming a negative feedback loop [9]. Accumulating evidence demonstrates a critical role for the circadian clock in regulating whole-body metabolism. A high-fat diet only induces obesity in mice if given ad libitum, but not only during the active phase [10]. Light at night increases body weight gain and the risk of glucose intolerance in mice fed with normal chow [11]. Glucose-induced insulin release is reduced in islet cells lacking the clock gene *Bmal1,* and these mice exhibit glucose intolerance [12]. Moreover, insulin itself can also regulate circadian rhythmicity, as mutated insulin receptors in neurons result in reduced circadian periods and signaling [13].

Under natural conditions, ambient light is the predominant environmental cue to regulate the body’s circadian rhythm. Light is received by intrinsically photosensitive retinal ganglion cells, and the information is transmitted to the SCN in mammals [14]. Aberrant light exposure can disrupt the circadian rhythm. Animals exposed to constant light can exhibit arrhythmic clock gene expression in the SCN and loss of circadian behavioral rhythms [15,16,17,18,19]. In addition, animals subjected to continuous exposure to light develop insulin resistance and experience more significant weight gain when compared to those in a standard 12 h light and 12 h dark cycle [17]. In the fetus, the SCN circadian clock becomes functional at around E14.5, and prenatal rhythmicity entrains to maternal rhythms [20,21,22]. The maternal metabolic health status or nutrient levels during pregnancy have been previously reported to have an additional impact on the metabolic health trajectory of the offspring [23,24,25,26,27,28,29]. For example, human studies and preclinical models show that offspring of dams who experienced malnutrition [23,30] or hypernutrition [31] during pregnancy have increased glucose intolerance in adulthood and susceptibility to type 2 diabetes (T2D). Shift work, particularly night shifts, disrupts biological rhythms, as well as sleep, and impacts pathways that increase the risk of metabolic disorders such as obesity and T2D [32]. In particular, the night shift has been shown to increase the risk of T2D in women [33]. Hence, we aimed to investigate whether disruption of circadian rhythms by ambient light using a “short day” paradigm starting in gestation might also contribute to the risk of metabolic diseases in the adult offspring.

To determine the impact of environmental circadian rhythm disruption on the regulation of glucose metabolism and homeostasis, we used an 8 h/8 h light/dark cycle to disrupt circadian rhythms from the beginning of gestation to adulthood and studied glucose tolerance and insulin sensitivity in adult offspring. We found that the offspring of dams exposed to SD displayed impaired glucose homeostasis and reduced sensitivity to insulin in a sexually dimorphic manner. These results highlight the significance of maintaining normal circadian rhythms during development and indicate that circadian rhythmicity is required for developing normal glucose metabolism and regulation in adulthood.

## 2. Materials and Methods

### 2.1. Animals

**Figure 1:**
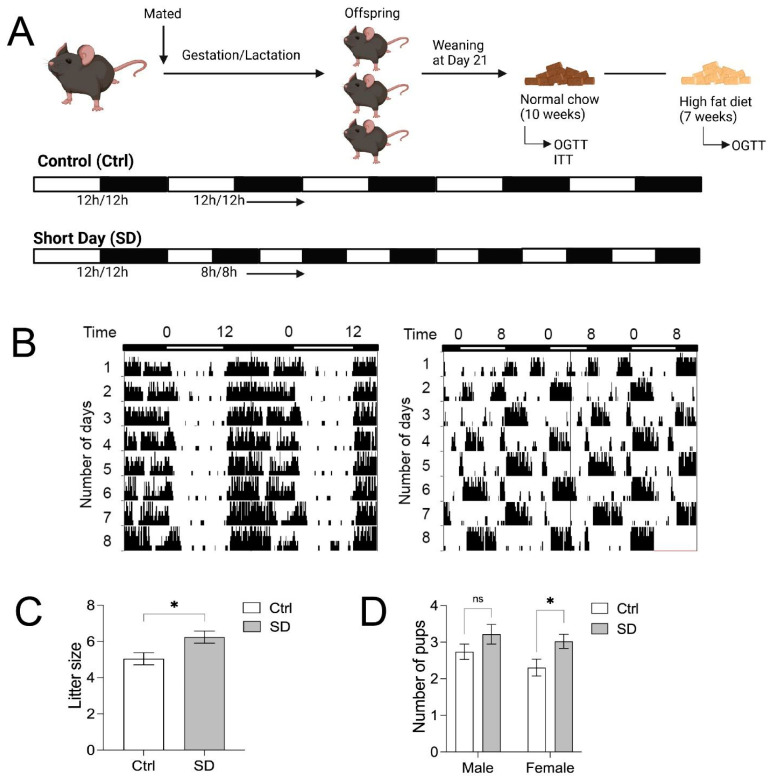
Experimental design. (**A**) Schematic timeline of the experiments in the current study. (**B**) Actograms of wheel-running activities of C57BL/6J mice under the 12 h/12 h (control, Ctrl and 8 h/8 h light/dark (short day, SD) cycles. (**C**) Litter size from Ctrl and SD dams. (**D**) Number of males and female pups born to Ctrl and SD dams. Statistical analysis was performed using an unpaired, two-tailed, Student’s *t*-test. * *p* ≤ 0.05, ns, not significant.

Male and female C57BL/6J breeder mice were purchased from the Jackson Laboratory (Stock No: 000664; RRID: IMSR_JAX:000664). All laboratory mice used for the study were maintained in the University of Minnesota, Duluth, animal facility with ad libitum access to the normal chow diet (ND, LabDiet 5053). The room temperature was maintained at 22 ± 1 °C with 35–45% humidity. All mouse colonies were kept in a 12 h/12 h light/dark (LD) cycle prior to the time of mating, where they were shifted to a short day (SD) cycle of 8 h/8 h LD (100 lux). Male and female breeders, six-weeks of age, were shifted to an 8 h/8 h LD for the entirety of gestation and lactation periods for the generation of SD offspring. SD offspring were also kept in the 8 h/8 h LD cycle throughout the duration of all experiments (Figure 1A). Control (Ctrl) mice were maintained and bred in the 12 h/12 h LD cycle. All Ctrl and SD offspring were fed a normal chow diet (NCD) for the first 7–10 weeks after weaning and later switched to a high-fat diet (HFD, Bio-Serv™ (Shanghai, China) S3282, 60% kcal from fat, Flemington, NJ, USA) for 7–8 weeks. Oral glucose tolerance tests (OGTT) and insulin tolerance tests (ITT) were performed at 7–10 weeks of age for NCD and at 14–16 weeks of age for HFD. All studies were approved by the Institutional Animal Care and Use Committee at the University of Minnesota (1606-33864A).

### 2.2. Mouse Wheel-Running Behavioral Recording

Mice were individually housed in cages equipped with running wheels and kept in the indicated light conditions. The wheel-running activities were recorded by the ClockLab software 6.1 (Actimetrics, Wilmette, IL, USA). Data were analyzed using the ClockLab Analysis software 6.1.

### 2.3. Metabolic In Vivo Mouse Studies

Body weight was collected from P5 to P142. Glucose and insulin measurements were assessed from tail vein blood using a handheld glucometer. The insulin tolerance test (ITT) was performed 2 h after light-on (Zeitgeber Time 2) for both Ctrl and SD mice following a 14 h fasting with blood glucose measurements at time 0 min prior to administration of 1.0 U/kg insulin (Humalog, Eli Lilly and Company, Indianapolis, IN, USA) in 0.9% saline i.p. and 30, 60, 90, and 120 min after insulin administration. Similarly, oral glucose tolerance tests (OGTT) were performed 1 h after light-on (Zeitgeber Time 1) by fasting mice for 13 h, and blood glucose was measured before and after a 2 g/kg glucose solution was administered via oral gavage. For the ITT test, Ctrl mice were starved from ZT12 to ZT2, and SD mice were starved from ZT4 to ZT2. For the OGTT test, Ctrl mice were starved from ZT12 to ZT1, and SD mice were starved from ZT4 to ZT1. Mice were given two weeks of recovery time between ITT and OGTT.

### 2.4. Protein Extraction and Western Blotting

Liver and skeletal muscle tissues were harvested at indicated time points, snap-frozen in dry ice, and stored at −80 °C until protein extraction. Protein extraction and Western blotting were performed as previously described [14]. In brief, tissues were homogenized in ice-cold lysis buffer (20 mM HEPES pH 7.5, 100 mM NaCl, 0.05% Triton X-100, 1 mM DTT, 5 mM Na-beta-glycerophosphate, 0.5 mM Na-vanadate, 1 mM EDTA, and protease inhibitors). The supernatant was collected, followed by a Bradford assay for protein quantification. Lysates were resolved on a 10% SDS PAGE gel and transferred to a PVDF membrane (Bio-Rad, Berkeley, CA, USA). Membranes were blocked with 5% nonfat dry milk and probed using primary antibodies for p-S6K1 (Thr389) antibody (1:1000, Cell Signaling Tech, Danvers, MA, USA, 9206, AB_2285392), S6K1 (1:2000, Cell Signaling Tech, 9202), p-ERK (Thr202/204) (1:1000, Cell Signaling Tech, 9106), ERK (1:2000, Santa Cruz, Shanghai, China, SC-93), p-AKT (Ser473) (1:1000, Cell Signaling Tech, 9271), and AKT (1:1000, Cell Signaling Tech, 9271). Blots were washed with 1× PBST and incubated in PBST (with 5% skim milk) with an HRP-conjugated secondary antibody (1:5000, GE Healthcare, Chicago, IL, USA, donkey anti-rabbit: NA931, AB_772210; donkey anti-mouse: NA934, AB_772206) for 1.5 h and wash repeated. Blots were incubated with an enhanced chemiluminescent reagent (Perkin Elmer, Waltham, MA, USA) to detect immunoreactivity and imaged using X-ray films. Protein bands were quantified using Adobe Photoshop 13.0 and normalized using the loading control glyceraldehyde-3-phosphate dehydrogenase (GAPDH).

### 2.5. Statistical Analysis

Statistical analysis was performed using the GraphPad Prism (version 10.1.1) software. An unpaired, two-tailed, Student’s *t*-test or 2-way ANOVA was used to compare group differences. Tukey’s multiple comparison test was used for post hoc tests.

## 3. Results

To investigate whether circadian rhythm disruption during development contributes to the risk of metabolic diseases in adulthood, we used a SD mouse model, in which an 8 h/8 h LD cycle was applied to disrupt circadian rhythms starting from the beginning of gestation throughout adulthood. We then evaluated glucose tolerance and insulin sensitivity in the adult mice. As demonstrated in Figure 1A, C57BL/6J breeding pairs were set up in the SD cycle and kept in SD throughout gestation. The offspring were kept in the SD cycle throughout the experiments. Control mice were kept in the standard 12 h/12 h LD cycle. We previously reported that rhythmic gene expression and behavioral rhythms are disrupted in SD adult mice [34]. As shown in Figure 1B, the control mice entrained to the standard 12 h/12 h LD cycle, whereas the SD mice entrained to the 8 h/8 h LD cycle. The litter size from SD dams was larger compared to the Ctrl group (Figure 1C), and statistical significance was observed in the number of female pups from SD mice (Figure 1D), indicating that the SD cycle did not impair the reproductivity of breeders.

**Figure 2:**
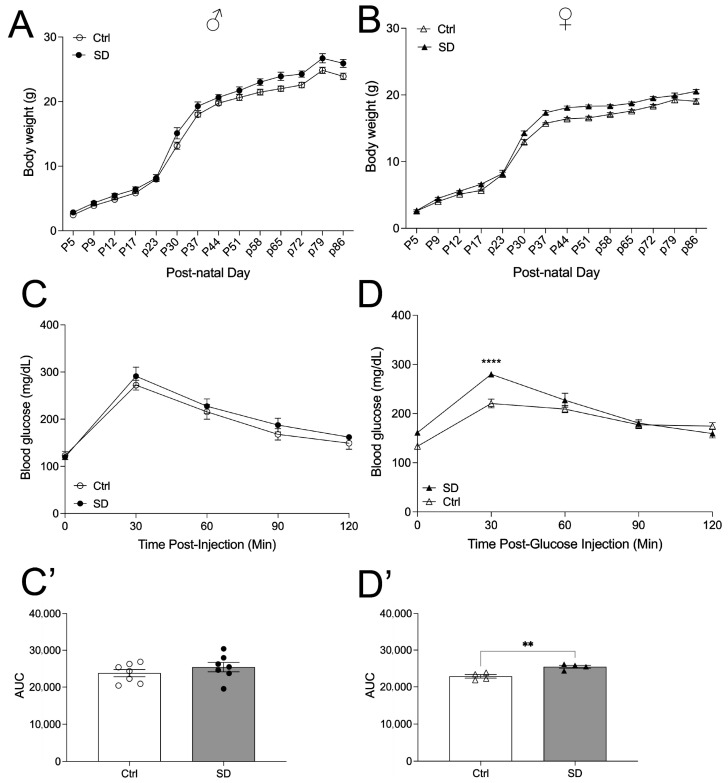
Female SD mice demonstrate impaired glucose tolerance on a normal chow diet (NCD). Male (circles) body weight (**A**) and female (triangles) body weight (**B**) of Ctrl and SD mice up to 86 days of postnatal age (males *n* = 7 and females n = 5–7). Oral glucose tolerance test (OGTT; 2 g/kg glucose) of males ((**C**), *n* = 6–7) and females ((**D**), n = 4) performed at 7–10 weeks of age. Area under curve analysis of OGTT males (**C’**) and females (**D’**). Statistical analysis performed using an unpaired, two-tailed, Student’s *t*-test or a 2-way ANOVA. ** *p* ≤ 0.01, **** *p* ≤ 0.0001.

**Figure 3:**
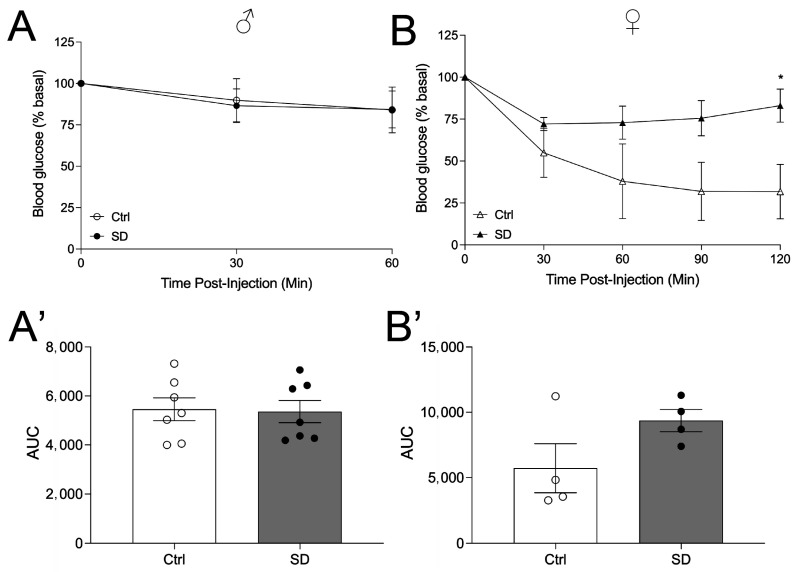
Female SD mice show impaired insulin sensitivity. The intraperitoneal insulin tolerance test (ITT; 1.00 U/kg insulin, i.p.) was performed at 7–10 weeks of age in males (circles) ((**A**), n = 7) and females (triangles) ((**B**), n = 4). AUC analysis of ITT in males (**A’**) and females (**B’**). Statistical analysis performed using an unpaired, two-tailed, Student’s *t*-test, or 2-way ANOVA. * *p* ≤ 0.05.

To assess potential changes in glucose metabolism in the SD mice, animals were tested for OGTT and ITT at the age of 10 weeks when fed a normal chow. Mice were then transferred to HFD for another 7 weeks and tested for OGTT again. Under a NCD, the body weight of the offspring was assessed, and a trend toward increased body weight was observed in both the male and female offspring of SD dams (Figure 2A,B). No changes in insulin levels were observed in male mice, but there was a trend of increased insulin levels in the female mice (*p* = 0.0506, Supplementary Figure S1A). OGTT was performed, and a significant glucose intolerance was observed in the female cohort but not in the male cohort (Figure 2C–D’), indicating that the SD cycle impairs glucose tolerance in a sexually dimorphic manner. To assess whether the glucose intolerance is due in part to altered insulin sensitivity in peripheral tissues, we performed ITT in male and female mice of 7–10 weeks of age. Male SD mice maintain normal insulin sensitivity relative to Ctrl mice (Figure 3A,A’). However, female SD mice demonstrated insulin resistance, and the level of blood glucose was higher at 120 min after insulin injection in the SD mice as compared to the Ctrl mice (Figure 3B,B’), consistent with the results of the OGTT.

Next, we sought to determine whether the insulin intolerance can be explained by altered insulin signaling responses in the skeletal muscle and liver in SD mice, as both tissues play a critical role in maintaining blood glucose homeostasis. Eight-week-old Ctrl and SD female mice were injected with insulin, and biochemical pathways of insulin signaling (phosphorylated AKT and S6K) were assessed 5 min after insulin injection.

**Figure 4:**
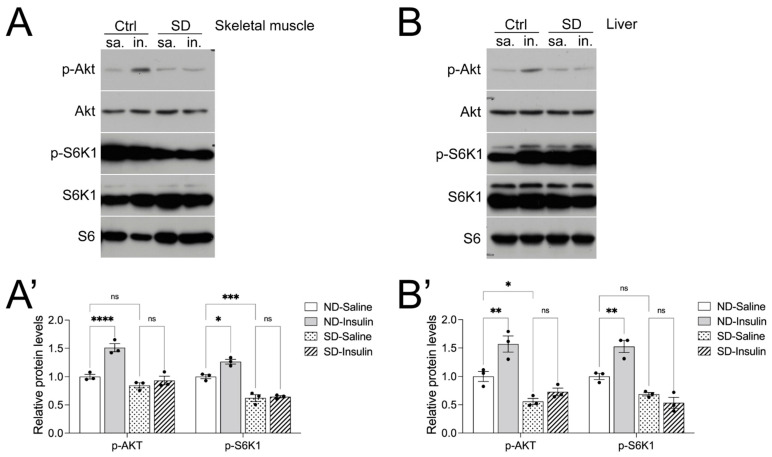
Female SD mice show a decreased insulin signaling response in the skeletal muscle and liver. (**A**) Representative image of Western blots of p-AKT, AKT, p-S6K1, and S6K1 of skeletal muscle lysates after stimulation with saline (sa) or insulin (in) with quantification of p-AKT and p-S6K1 in (**A’**). (**B**) Representative image of Western blots of p-AKT, AKT, p-S6K1, and S6K1 of liver lysates after stimulation with sa or in with quantification of p-AKT and p-S6K1 in (**B’**). Skeletal muscle and liver tissue were harvested from 8-week-old female SD mice 5 min after administration of 1.0 U/kg insulin i.p. The levels of p-AKT and p-S6K1 were normalized based on the levels of total AKT and S6K1. Ribosomal proteins S6 (S6) and β-actin were used as loading controls. Statistical analysis was performed using a 2-way ANOVA. * *p* ≤ 0.05, ** *p* ≤ 0.01, *** *p* ≤ 0.001, **** *p* ≤ 0.0001, ns, not significant.

In the Ctrl animals, phosphorylated AKT (p-AKT) and S6K1 in skeletal muscle and the liver were significantly increased in response to insulin treatment, as expected (Figure 4A,B). However, the levels of p-AKT and p-S6K1 were not altered by insulin in the SD mice (Figure 4A,B), indicating an impaired insulin signaling response in the SD animals. Ribosomal proteins S6 (S6) and β-actin were used as loading controls. No changes in total S6K or AKT protein levels were observed between the groups tested. We also assessed the levels of phosphorylated ERK1/2, but no differences were observed between saline and insulin treatment within groups (Supplementary Figure S2A), indicating that insulin specifically activated the AKT/mTORC1 pathway but not the ERK MAPK pathway at this time point.

**Figure 5:**
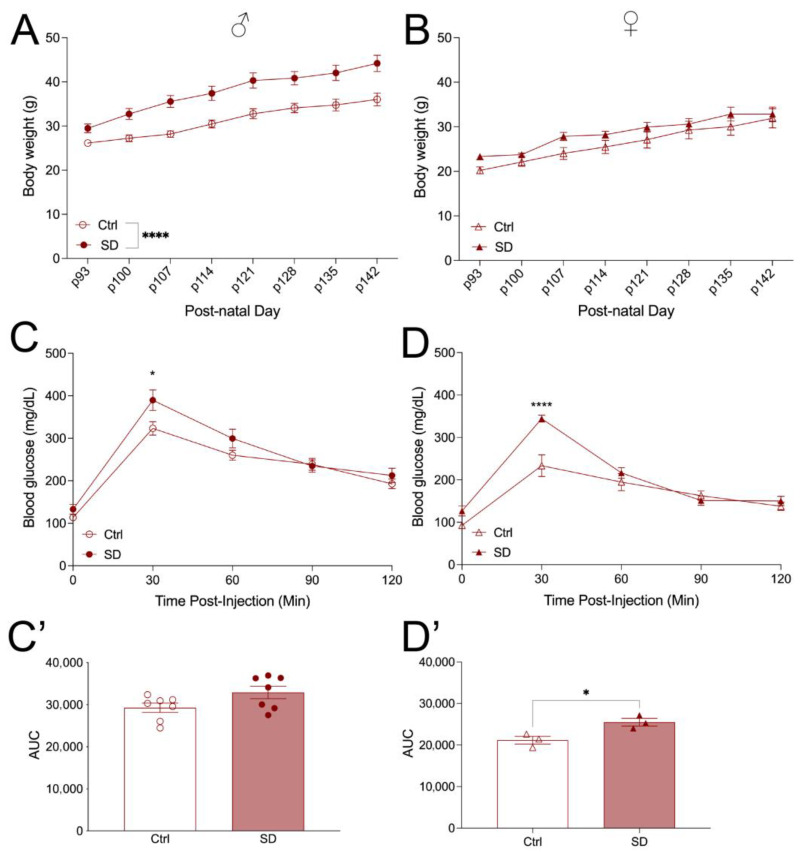
Both male and female SD mice demonstrate glucose intolerance on a high-fat diet. Male (circles) body weight (**A**) and female (triangles) body weight (**B**) of Ctrl and SD mice (males n = 7, females n = 7). Oral glucose tolerance test (OGTT; 2 g/kg glucose) of males ((**C**), n = 7) and females ((**D**), n = 3) performed at 14–16 weeks of age. AUC analysis of OGTT males (**C’**) and females (**D’**). Statistical analysis was performed using an unpaired, two-tailed, Student’s *t*-test or 2-way ANOVA. * *p* ≤ 0.05, **** *p* ≤ 0.0001.

Under the metabolic challenge of a HFD (60% kcal), as expected, both male and female Ctrl and SD mice gained weight during treatment (Figure 5A,B). However, it is notable that SD animals gained more body weight than their Ctrl counterparts. Interestingly, the male SD mice demonstrated significantly higher weight gain relative to the control. The female SD mice showed a trend of more weight gain than control, but the difference was not significant (Figure 5A,B). Notably, under HFD, both male and female SD displayed glucose intolerance relative to their respective controls (Figure 5C,D), as demonstrated in the OGTT test. Compared to the control, both male and female SD mice displayed elevated glucose levels 30 min after glucose injection. Only the female SD displayed a significant change in the area under the curve (Figure 5C’,D’). The elevated glucose level at the 30 min time point in the OGTT could suggest a pancreas defect, but no significant changes in insulin levels were observed in either male or female mice under non-fasting and fasting conditions (Supplementary Figure S1B,C). Together, these results indicate that HFD further impaired glucose homeostasis in both male and female SD mice.

## 4. Discussion

Disruption of circadian rhythms by aberrant light exposures may have long-lasting health consequences in adulthood. In the current study, we focused on the effects of circadian disruption on metabolism and showed that disruption of circadian rhythms is sufficient to impair glucose homeostasis, namely, glucose tolerance and insulin sensitivity in female SD mice under a NCD. Glucose intolerance is often associated with perturbed glucose clearance, insulin resistance in peripheral tissues, and reduced insulin secretion by pancreatic beta-cells. Molecularly, glucose intolerance can be explained in part by impaired insulin signaling in both the skeletal muscle and hepatic tissues. When insulin binds to its receptor, autophosphorylation of tyrosines located on the intracellular subunit leads to phosphorylation of insulin receptor substrate-1 and subsequently activation of the AKT/PKB signaling pathway. AKT is an upstream regulator of mTORC1, a major nutrient sensor, and a crucial component in insulin sensitivity with its role in GLUT4 translocation in skeletal muscle [35]. In the current paper, we demonstrated that under the metabolic challenge of a HFD, both male and female SD mice displayed increased glucose intolerance and body weight gain. Gaining weight following a HFD is not unexpected, as consumption of HFD typically leads to obesity and the deposition of fat in unexpected areas such as the liver. This pathological state is often associated with hyperglycemia and hyperinsulinemia, both of which adversely impact glucose homeostasis.

Circadian rhythm disruption is associated with impaired glucose homeostasis and T2D. Disruptions of the normal LD cycle in wild-type mice are analogous to shift work disruptions in humans. Evening shift work is associated with approximately 75% increased odds of developing gestational diabetes (GDM) [36]. GDM is recognized for its association with maternal obesity, glucose intolerance, and the birth of large gestational-age infants. This is attributed to the heightened transfer of glucose from the mother to the fetus, leading to an increase in fetal insulin levels that alters the infant’s growth trajectory. Consequently, in-utero exposure during GDM has been linked to adverse effects on fetal and adult offspring metabolic health, including an elevated susceptibility to various diseases later in life, such as obesity, type 2 diabetes (T2D), and hypertension [37]. Various diets and time-feeding restrictions can directly impact circadian rhythm by altering the expression of core clock genes. For example, high-fat diets disrupt circadian rhythms [38,39], and calorie restriction can positively restore them [40]. Interestingly, time-restricted feeding regulates circadian rhythm to prevent metabolic diseases such as those induced by a HFD [40]. At the molecular level, tissue-specific disruption of any clock genes (i.e., CLOCK and BMAL1) regulating circadian rhythm accelerates the development of diabetes through pancreatic beta-cell loss and dysfunction [12,41]. In the pancreas, time-restricted feeding prevents the deleterious metabolic effects of circadian disruption through epigenetic control of beta cell function [42]. However, the applicability of these conclusions to pregnant dams is currently unknown. The potential impact on offspring born to dams whose metabolic effects are disrupted by circadian disruption and subsequently restored through time-restricted feeding remains unclear and warrants further investigation.

We previously reported that PER1, PER2, and BMAL1 circadian oscillations are lost in the SCN and the hippocampus of the SD model [34]. These key clock genes are also lost in peripheral tissues when circadian rhythms are disrupted [43]. SD mice show arrhythmic wheel-running behavior under constant darkness. Hence, we speculated that the SD cycle disrupts systematic circadian gene expression and circadian synchronization in different tissues, including the skeletal muscle and the liver. A number of environmental circadian disruption models have reported higher body weight gain, which is consistent with the higher body gain phenotype of SD mice under a HFD. It is important to note that HFD in mice leads to changes in the period of the locomotor activity rhythm and alterations in the expression and cycling of clock genes and clock-controlled genes involved in fuel utilization in the hypothalamus, liver, and adipose tissue [38]. In human studies, short-term circadian disruption, or “misalignment”, such as shift work, induces a significant decrease in muscle insulin sensitivity [44] and glucose tolerance [45,46]. In addition, the circadian rhythm can be regulated by insulin itself. In the context of a HFD, mice typically exhibit insulin resistance, leading to metabolic dysfunction that can further disrupt circadian rhythms [47]. Furthermore, the consumption of a HFD is known to disrupt the normal feeding and fasting cycles throughout the day, resulting in a loss of circadian rhythm [48]. Consequently, it is not surprising that the SD mice demonstrated exacerbated insulin resistance and glucose intolerance under the HFD condition compared to the NCD. Other studies have shown that circadian rhythm disruption can specifically induce glucose intolerance mainly by lowering insulin sensitivity, not by affecting pancreatic beta-cell function [49].

In the current study, we showed that circadian disruption in utero and early in life affects metabolism in a sexually dimorphic manner: female but not male SD mice exhibited significant impairments in glucose tolerance under a NCD. C57BL6/6J male mice typically exhibit impaired glucose intolerance, insulin resistance, and greater beta-cell mass compared to females when subjected to a HFD [50]. Under a NCD, C57BL6/6J males display glucose intolerance compared to age-matched female controls, in part due to impaired insulin tolerance [51]. Intriguingly, our data indicate that female SD mice fare worse than males in terms of glucose homeostasis in a NCD. Our study conducted under a NCD revealed a notable difference in the 30 min time point of the female OGTT. Furthermore, there seems to be a gradual increase at the 60 min time point, although it lacks statistical significance. The underlying explanation for these findings remains uncertain. During the initial phase of the glucose tolerance test (5–10 min), insulin secretion reaches its peak and subsequently aids in reducing glucose levels. Conversely, the latter half of the glucose curve indicates peripheral insulin resistance. Based on this, we hypothesize that the total insulin secretion at the 30 min mark might be altered in SD female mice, potentially contributing to delayed glucose clearance. In the future, we will evaluate beta-cell function at the islet level to better understand and differentiate these observed changes. Furthermore, the disparity between insulin sensitivity in SD female mice and their capacity to clear glucose highlights the importance of investigating beta-cell function. However, since fasting insulin levels were not found to be altered between SD and Ctrl mice, it is unlikely that beta-cell mass was affected. Consequently, we deem it essential to evaluate insulin secretion in SD mice using dynamic perifusion, which represents a pressing area of investigation for our laboratories in the near future.

The influence of disrupted circadian rhythm on sexual dimorphism has also been observed in human studies, particularly regarding energy regulation [52]. In particular, females exhibit lower levels of leptin, a hormone involved in energy balance [52]. Consequently, females are generally more vulnerable to disturbances in energy homeostasis. Therefore, the divergent effects of circadian disruption on male and female energy regulations can potentially contribute to the sexual dimorphism observed in our findings. Further studies are required to identify specific mechanisms underlying the sex-specific metabolic abnormalities of the SD mice. Based on our findings, we acknowledge one limitation in our study in which SD mice did not undergo a fasting period according to their SD cycle, unlike Ctrl mice, who followed a standard 12 h/12 h LD cycle and were fasted for 13 h before the OGTT experiments. This difference meant that SD mice were fasted for 81% of their day, while Ctrl mice were fasted for only 54% of their total day. Fasting differences were considered in our experiments, as C57BL/6J mice mostly feed during the dark phase [53]. Therefore, we ensured both groups were fasted for a complete dark phase. We ensured that both groups fasted for a complete dark phase before the ITT or OGTT test. Both groups were starved for a period spanning over a complete dark phase. This limitation should be considered when interpreting experiments with 13 h fasting (OGTT and ITT). Furthermore, it is worth noting that ZT2 is not the optimal time in terms of insulin or glucose sensitivity for mice [54]. However, our experimental design ensured that both the SD and Ctrl groups were subjected to the inactive phase during data collection. Since both the SD and Ctrl mice experiments were performed at the same time, during the inactive phase, there are fewer differences to discern between groups.

## 5. Conclusions

Disruption of circadian rhythms across lifespans by ambient light leads to metabolic dysfunction in adult mice. Disruption of circadian rhythms by the 8 h/8 h LD cycle increases glucose intolerance and insulin resistance in female mice. Molecularly, glucose intolerance can be explained in part by insulin resistance and perturbed insulin signaling in both the skeletal muscle and hepatic tissues. Increased susceptibility to obesogenic challenges and metabolic dysfunctions is found in both male and female mice when circadian disruption is coupled with HFD. Altogether, these results demonstrate that environmental disruption of circadian rhythms across lifespans increases the risk of obesity and type 2 diabetes in adulthood.

## Data Availability

The data presented in this study are available upon request from the corresponding authors due to privacy.

## Supplementary Materials

The following supporting information can be downloaded at: https://www.mdpi.com/article/10.3390/metabo14020126/s1, Figure S1: Non-fasting and fasting insulin concentration of Ctrl and SD mice; Figure S2: Western blots of p-ERK and ERK.
