# Supplementary material for: Circadian Disruption across Lifespan Impairs Glucose Homeostasis and Insulin Sensitivity in Adult Mice
Source: Metabolites. 2024 Feb 16;14(2):126. doi: 10.3390/metabo14020126 (PMC10892663; doi:10.3390/metabo14020126)
Supplement: Supplementary file 1 [file metabolites-14-00126-s001.zip › metabolites-2782581-supplementary.pdf]

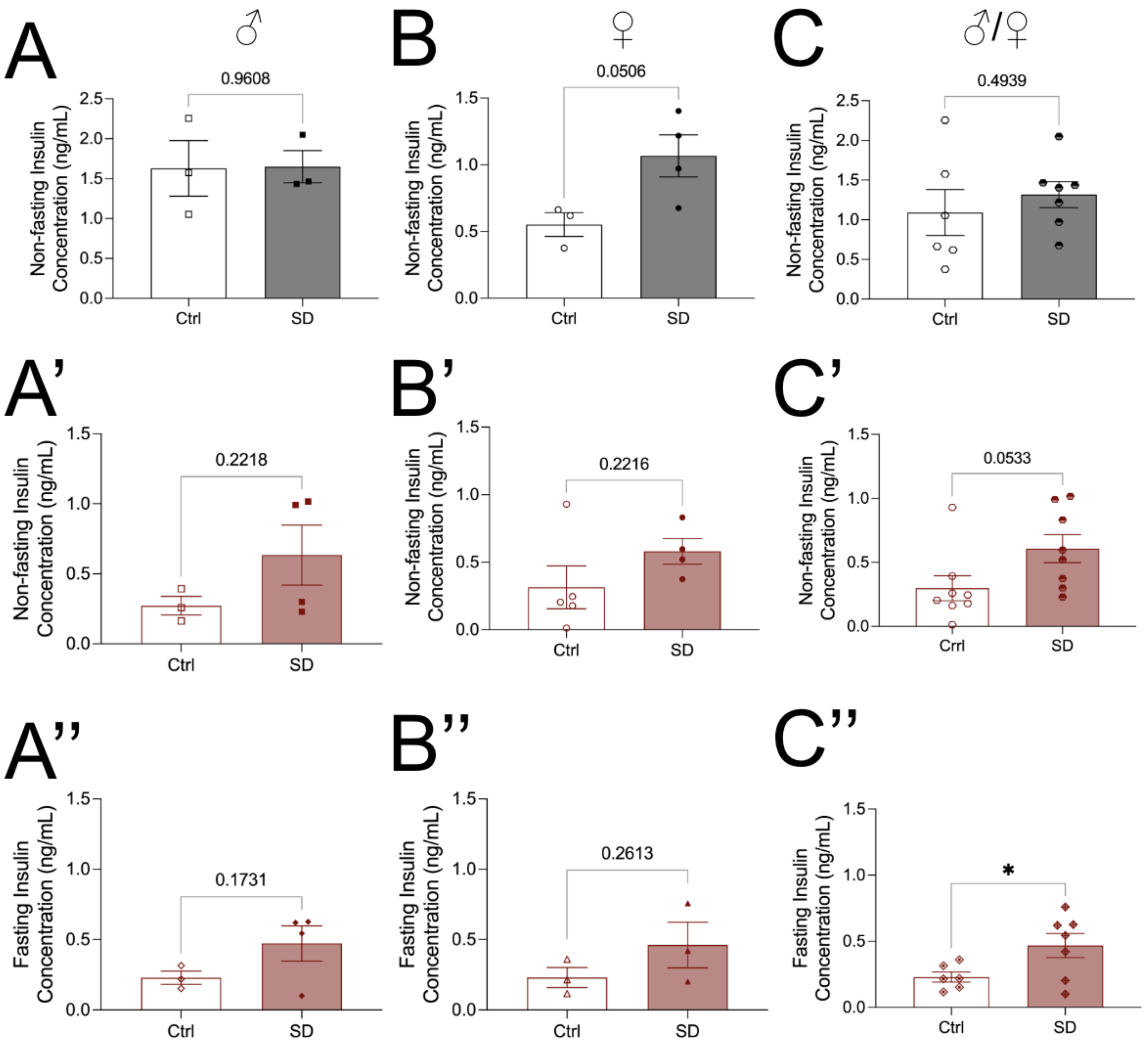

Figure S1. Non-fasting and fasting insulin concentration of Ctrl and SD mice. Non-fasting insulin levels of Ctrl (n= 3) and SD (n = 3-4) male mice in NCD (A). Non-fasting (A′) and fasting (A′′) insulin levels of Ctrl (n= 3) and SD (n = 3-4) male mice in HFD. Non-fasting insulin levels of Ctrl (n= 3) and SD (n = 4) female mice in NCD (B). Non-fasting (B′) and fasting (B′′) insulin levels of Ctrl (n= 3-5) and SD (n = 3-4) female mice in HFD. Combined male and female non-fasting insulin concentration under NCD (C). Combined male and female non-fasting (C′) and fasting (C′′) insulin concentration under HFD. Statistical analysis performed using unpaired, two-tailed, student T-Test. *P ≤ .05, ****P ≤ .0001.

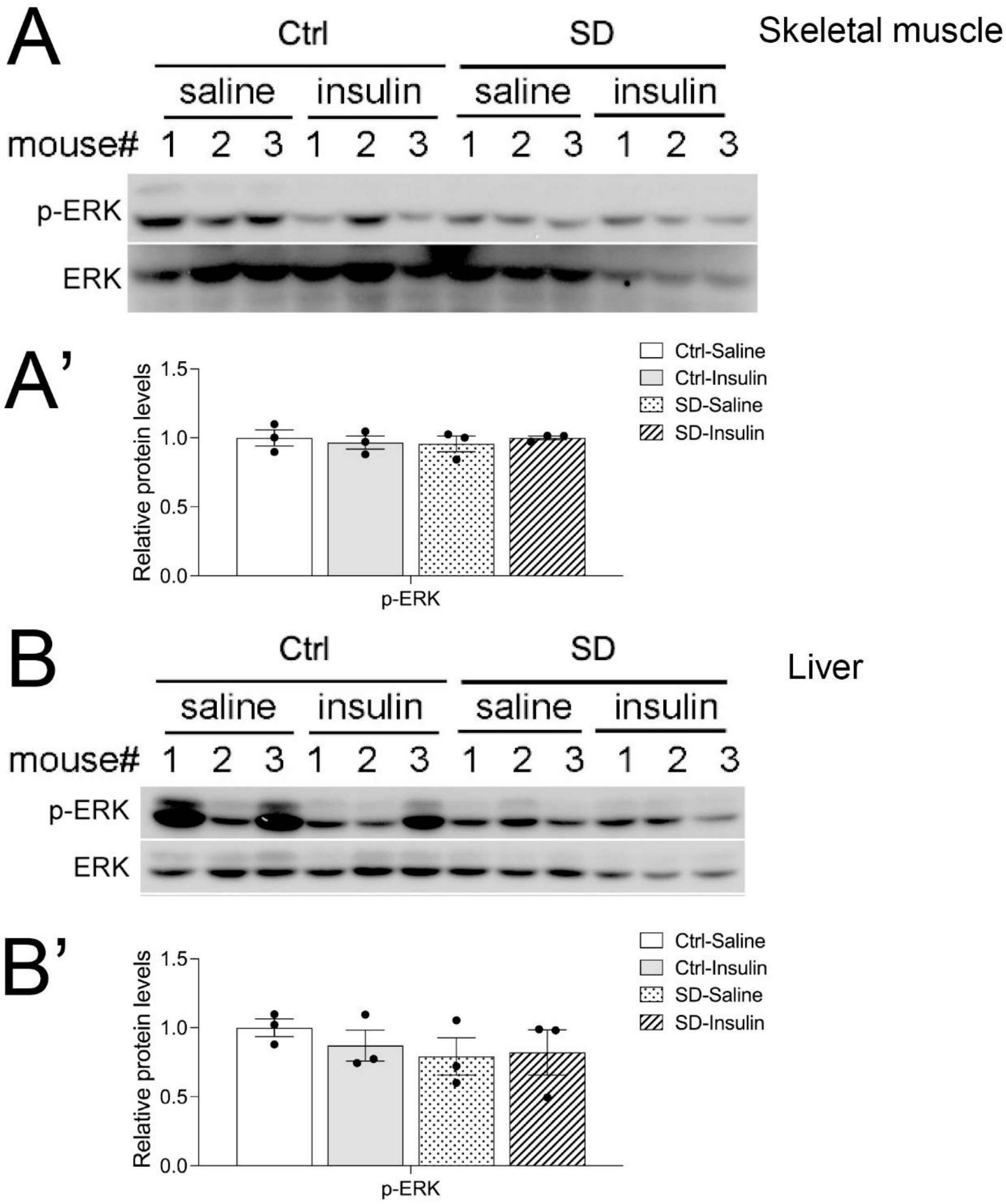

Figure S2. Western blots of p-ERK and ERK. Blots from lysates of skeletal muscle (A) with quantification (A') from female SD mice. Tissues were harvested from 8-week-old female SD mice 5 min after *i.p.* administration of 1.0 U/kg insulin. Statistical analysis performed using unpaired, two-tailed, Student's t-test.
